# Supplementary material for: Comparative Mitogenomics Reveals Cryptic Species in Sillago ingenuua McKay, 1985 (Perciformes: Sillaginidae)
Source: Genes (Basel). 2023 Nov 4;14(11):2043. doi: 10.3390/genes14112043 (PMC10671150; doi:10.3390/genes14112043)

Table S1. Nucleotide composition and skews for mitochondrial 13 PCGs, tRNA, rRNA and control region (CR) in the genus *Sillago*.

**Species**	**T (%)**	**C (%)**	**A (%)**	**G (%)**	**AT - skew**	**GC - skew**	**Species**	**T (%)**	**C (%)**	**A (%)**	**G (%)**	**AT - skew**	**GC - skew**
***S. ingenuua* A**	26.1	29.2	28.4	16.3	0.041	-0.284	***S. indica***	27.3	29.0	25.5	18.1	-0.033	-0.231
PCGs	28.3	30.3	25.8	15.7	-0.045	-0.318	PCGs	29.6	30.1	22.6	17.8	-0.133	-0.256
tRNA	26.8	21.4	26.8	25.0	0.001	0.079	tRNA	26.7	21.6	26.8	24.8	0.002	0.069
rRNA	21.3	25.5	32.4	20.8	0.206	-0.101	rRNA	21.7	25.8	30.5	22.0	0.169	-0.080
CR	32.7	18.8	32.7	15.8	0.000	-0.088	CR	30.3	22.4	30.9	16.5	0.010	-0.151
***S. ingenuua* B**	25.9	29.3	28.5	16.4	0.048	-0.282	***S. japonica***	27.5	27.9	26.4	18.3	-0.021	-0.208
PCGs	28.1	30.3	26.0	15.7	-0.038	-0.317	PCGs	30.0	29.0	23.2	17.8	-0.127	-0.239
tRNA	26.7	21.4	27.0	25.0	0.005	0.078	tRNA	26.4	21.6	27.5	24.4	0.020	0.060
rRNA	21.1	25.6	32.2	21.2	0.208	-0.094	rRNA	21.9	24.4	31.3	22.4	0.177	-0.044
CR	30.6	20.3	33.4	15.6	0.044	-0.131	CR	29.1	22.2	33.2	15.4	0.066	-0.179
S. aeolus	26.0	29.7	25.5	18.8	-0.009	-0.226	***S. sihama***	26.5	29.3	25.8	18.3	-0.012	-0.230
PCGs	28.1	30.9	22.6	18.4	-0.107	-0.254	PCGs	28.8	30.4	22.7	18.0	-0.118	-0.256
tRNA	26.8	21.6	26.6	25.1	-0.004	0.076	tRNA	26.5	21.8	27.0	24.8	0.010	0.065
rRNA	20.7	26.2	30.8	22.3	0.196	-0.081	rRNA	20.8	26.4	30.2	22.5	0.183	-0.080
CR	28.8	23.5	30.4	17.3	0.026	-0.154	CR	27.7	23.2	32.8	16.3	0.085	-0.175
S. asiatica	26.6	29.4	25.4	18.7	-0.023	-0.222	***S. sinica***	26.1	28.9	25.2	19.7	-0.018	-0.190
PCGs	28.8	30.4	22.3	18.5	-0.126	-0.244	PCGs	28.0	30.0	22.5	19.4	-0.109	-0.215
tRNA	26.5	22.2	27.3	24.1	0.016	0.042	tRNA	26.7	21.6	26.8	24.8	0.002	0.069
rRNA	20.9	25.9	31.0	22.2	0.195	-0.075	rRNA	21.9	24.8	30.2	23.1	0.159	-0.037
CR	29.7	23.2	30.0	17.0	0.004	-0.153	CR	28.4	22.7	29.8	19.0	0.025	-0.089

Figure S1. Inferred secondary structures for the 22 tRNA genes of *Sillago ingenuua* (tRNA genes of *S. ingenuua* A were showed and mutation sites of *S. ingenuua* B were marked).

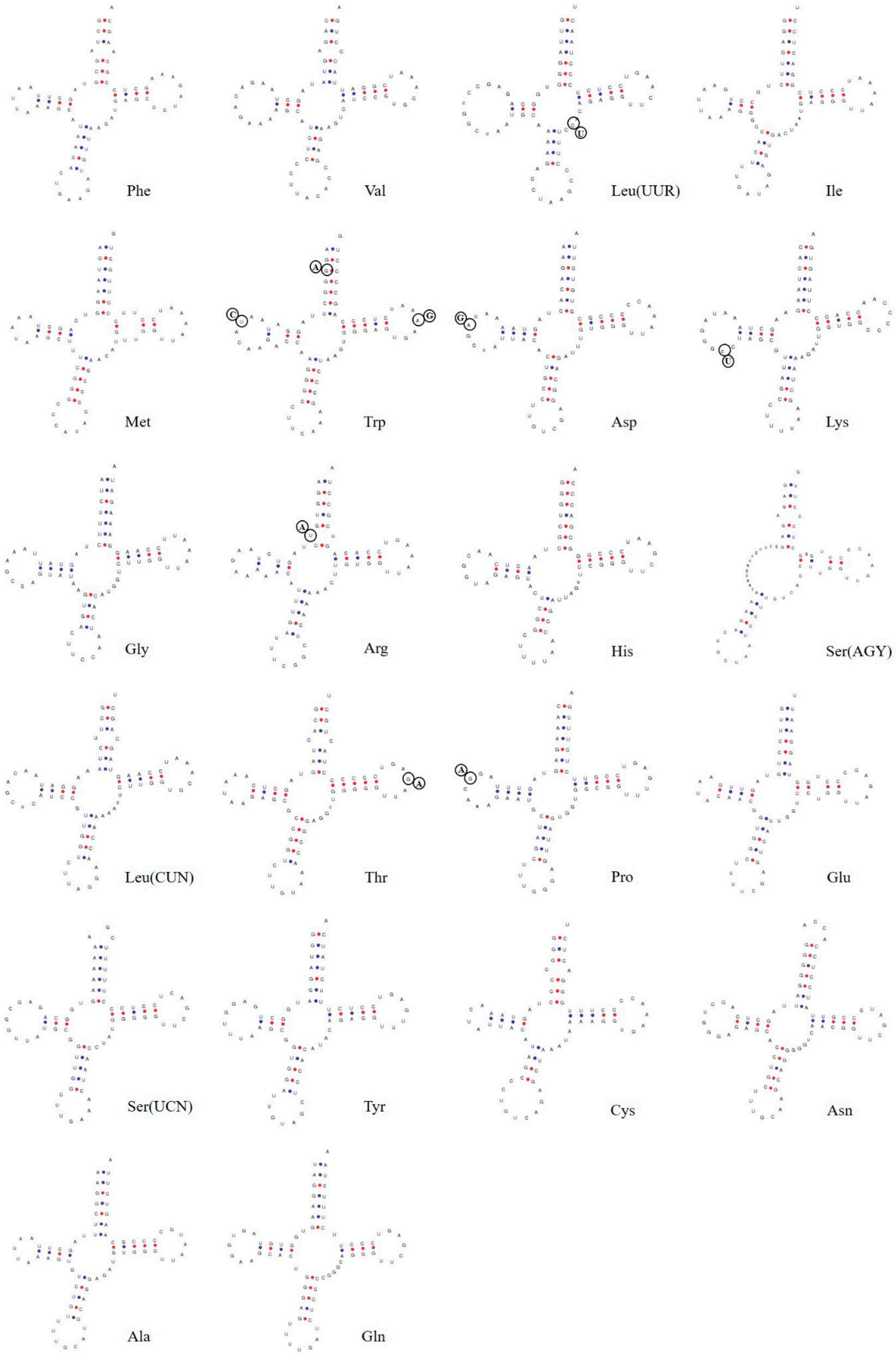

Supplement: Supplementary file 1 [file genes-14-02043-s001.zip › genes-2673146-supplementary.pdf]
